# Expert opinion about laser and intense pulsed light (IPL)-induced leukoderma or vitiligo: a cross-sectional survey study

**DOI:** 10.1007/s00403-023-02611-8

**Published:** 2023-03-25

**Authors:** Nicoline F. Post, Noah X. Van Broekhoven, Annelies Lommerts, Jung M. Bae, Marcel W. Bekkenk, Caio C. Silva de Castro, Viktioria Eleftheriadou, Samia Esmat, Khaled Ezzedine, Nanja van Geel, Iltefat Hamzavi, Giovanni Leone, Amit G. Pandya, Thierry Passeron, Michelle A. Rodrigues, Julien Seneschal, Steven Th’ng, Albert Wolkerstorfer

**Affiliations:** 1 Department of Dermatology, Amsterdam University Medical Centers, Meibergdreef 9, 1105 AZ Amsterdam, The Netherlands; 2 Department of Dermatology, St. Vincent’s Hospital, Seoul, South Korea; 3 Department of Dermatology, Pontifical Catholic University of Paraná, Curitiba, Brazil; 4 Department of Dermatology, The Royal Wolverhampton NHS Trust, Wolverhampton, UK; 5 Department of Dermatology, Cairo University, Giza, Egypt; 6 Department of Dermatology, Université Paris-Est Créteil Val de Marne–Université Paris, Paris, France; 7 Department of Dermatology, Ghent University Hospital, Ghent, Belgium; 8 Department of Dermatology, Henry Ford Health System, Detroit, MI USA; 9 Dermatology Department, Israelite Hospital, Rome, Italy; 10 Department of Dermatology, Palo Alto Foundation Medical Group, Sunnyvale, CA USA; 11 Department of Dermatology and INSERM U1065, University Hospital of Nice, Nice, France; 12 Chroma Dermatology, Pigment and Skin of Colour Centre, Victoria, Australia; 13 Department of Dermatology and Pediatric Dermatology, University Hospital, Bordeaux, France; 14 Department of Dermatology, Skin Research Institute of Singapore, Singapore, Singapore

**Keywords:** Vitiligo, Leukoderma, Koebner phenomenon, Laser, IPL

## Abstract

Vitiligo patients may desire laser hair removal, skin rejuvenation, vascular treatments, and other laser or intense pulsed light (IPL) assisted treatments. However, there is a risk of inducing new depigmented patches (Koebner phenomenon). In absence of guidelines on the safe use of laser or IPL in vitiligo patients, dermatologists tend to be reluctant to administer these treatments. The aim of this survey study was to provide an estimation of the occurrence and related risk factors of laser/IPL-induced leukoderma or vitiligo. A cross-sectional survey study was performed among 15 vitiligo experts from 11 countries, with 14 questions about affected patients, involved laser/IPL treatments and the physicians’ approach. In a total of 11,300 vitiligo patients, laser/IPL-induced leukoderma or vitiligo was reported in 30 patients (0.27%). Of these, 12 (40%) patients had a medical history of vitiligo and seven (58%) of these patients had stable (> 12 months) vitiligo before the treatment. Most frequently reported were hair removal procedures and localization of the face and legs. Side effects like blistering, crusting, and erosions occurred in 56.7% of the cases. These vitiligo experts based their advice on the risk of the laser treatment on stability of the vitiligo (43%) and activity signs (50%), and 50% discuss the risks before starting a laser treatment. Relevant activity signs are the Koebner phenomenon (57.1%), confetti-like lesions (57.1%) and hypochromic borders (50%). Laser-induced leukoderma or vitiligo is an uncommon phenomenon. Remarkably, a minority had a medical history of vitiligo of which 58% were stable. Consequently, most cases could not have been prevented by not treating vitiligo patients. However, a majority had laser/IPL-induced skin damage. Therefore, caution is advised with aggressive settings and test-spots prior to the treatment are recommended. This study showed significant variation in the current recommendations and approach of vitiligo experts regarding laser/IPL-induced leukoderma or vitiligo.

**Supplementary Information:**

The online version contains supplementary material available at 10.1007/s00403-023-02611-8.

## Introduction

Vitiligo is a pigmentary disorder characterized by sharply demarcated depigmented macules with a potentially high social and psychological burden [1]. Vitiligo patients may desire laser hair removal, skin rejuvenation, vascular treatments and other laser or intense pulsed light (IPL) assisted treatments. However, by causing trauma to the skin, there’s a risk of inducing new depigmented patches called the Koebner phenomenon [2]. Various kinds of skin injury may induce the Koebner phenomenon in vitiligo patients, including physical injury, mechanical trauma, chemical burns, thermal burns, allergic reactions, chronic pressure and therapeutic interventions [3]. Vitiligo patients with a history of Koebner phenomenon tend to have higher body surface area (BSA) involvement, an earlier age at onset of vitiligo and elevated risk of further depigmentation despite treatment [4]. Furthermore, a systematic review showed evidence for the Koebner phenomenon in relation to active vitiligo [5]. In the absence of any guidelines on the safe use of laser or IPL in vitiligo patients, dermatologists tend to be reluctant to administer these treatments. To date, only anecdotal evidence from six case reports is available on laser or IPL induced vitiligo [6]. The aim of this international survey study among vitiligo experts was to provide an estimation of the occurrence and related risk factors of laser/IPL-induced leukoderma or vitiligo.

## Materials and methods

In June 2021, a cross-sectional [7] survey study was performed with 15 dermatologists from 11 countries*.* The selection of participating dermatologists was based on scientific and clinical expertise in vitiligo and aimed at an international representation across several continents and cultures.

We developed an online questionnaire in Google Forms. 14 questions were carefully formulated and pretested by dermatologists (AL, AW and MB). Questions were divided into 3 sections: the affected patients, the involved laser/IPL treatments and the physicians’ approach (*supplementary file appendix 1*). Dermatologists were asked to report every case of laser-induced leukoderma or vitiligo over the past year and to report how many vitiligo patients they approximately encountered (face to face) over the past year. Laser/IPL-induced leukoderma or vitiligo was defined as a progressive depigmentation in a laser/IPL treated area (in patients with or without a history of vitiligo). Q-Switched laser treatments with the intention to induce depigmentation were excluded. If a dermatologist reported a case, they were asked about the likelihood of the leukoderma or vitiligo being caused by laser or IPL, type of procedure, medical history of the patient, stability of vitiligo (if present in medical history), interval between treatment and onset of leukoderma or vitiligo, localization of leukoderma or vitiligo, and visible side effects. The two final questions involved the dermatologists’ approach when a vitiligo patient desires a laser treatment and which activity signs the dermatologists consider relevant in the advice on the risk of laser treatment. After 2 weeks, one reminder was sent to the dermatologists who had not yet responded. According to recommendations of the CROSS-Checklist all answers were checked for clarity and inconsistencies and if needed clarified by the concerned dermatologist.

Single stage sampling was used since all the data were extracted from the questionnaire during the response period of 8 days. As there are no data available about the incidence of laser-induced leukoderma or vitiligo, an upfront sample size calculation was not possible. The occurrence of laser/IPL-induced leukoderma or vitiligo was calculated by all the potential laser/IPL-induced leukoderma or vitiligo patients that were reported, divided by all the vitiligo patients they approximately had encountered (face to face) over the past year to calculate the sample size. The data set was extracted from Google Forms into an Excel format and checked for irregularities by NP, and NB. Then, the Excel sheet was imported into IBM SPSS statistics 26 to provide descriptive statistics. No other statistical tests have been performed.

Ethics approval for this study was not necessary, as patients were not directly involved and the collected patient-related data were completely anonymous. The link to the questionnaire was sent directly to the vitiligo experts after approval was given to partake in the study. Furthermore, the questionnaire could only be filled in if accompanied by an e-mail address which was checked to prevent multiple participation.

## Results

Fourteen of the 15 invited vitiligo experts participated in the survey (93% response rate). One participant was unable to respond due to time constraints. Vitiligo experts participated from the following countries: Australia, Belgium, Brazil, Egypt, France, Italy, Singapore, South Korea, The Netherlands, United Kingdom, and United States of America. One answer was incompatible with the following questions and was therefore clarified by the concerned dermatologist. Together the dermatologists had encountered (face to face) 11,300 vitiligo patients over the past year (May 2020–May 2021). Laser/IPL-induced leukoderma or vitiligo was reported in 30 patients (0.27%) as likely to very likely to be laser/IPL-induced leukoderma or vitiligo. Only 12 (40%) cases already had vitiligo prior to the treatment and seven (58%) of these patients were stable before the treatment. 17% had an active vitiligo over the past 12 months. For the other 25%, it was unknown whether their vitiligo was active or stable.

### Cases of laser and IPL induced vitiligo

**Fig. 1:**
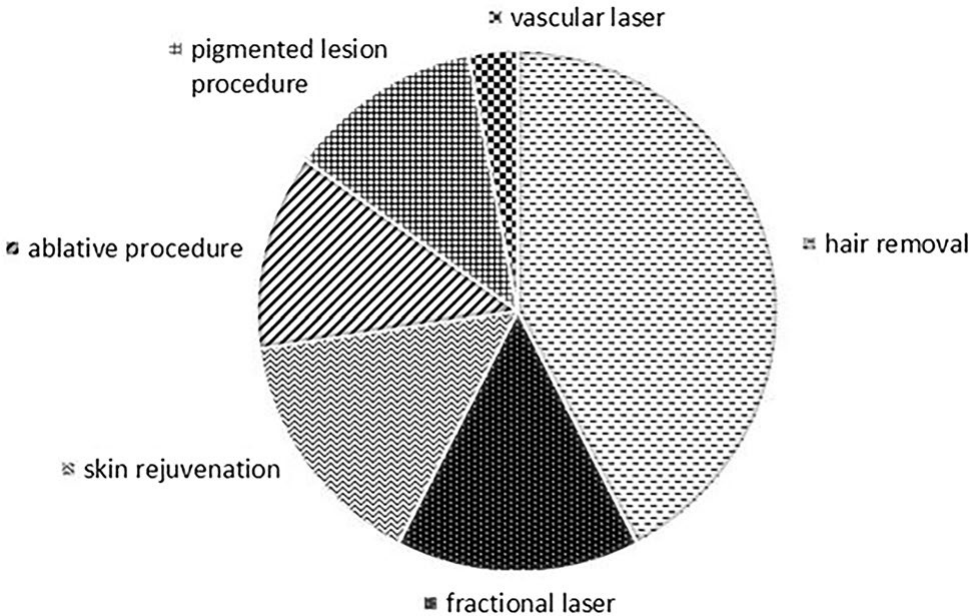
Frequencies of laser or IPL procedures that caused laser-induced vitiligo

Out of the 30 reported cases, 3 cases were patients with Fitzpatrick skin type 2 (10%), 14 with skin type 3 (46.7%), and 13 with skin type 4 (43.3%). This corresponds with the overall skin type distribution of the 11,300 vitiligo patients—Fitzpatrick skin type 1 (0.4%), 2 (15.7%), 3 (46.6%), 4 (34.7%), 5 (2.2%), and 6 (0.3%). In Fig. 1, the frequencies of laser-induced leukoderma or vitiligo per type of procedure is shown. Hair removal procedures account for 46.7% of the laser-induced vitiligo cases. Furthermore, fractional laser procedures and skin rejuvenation both account for 16.7% of the laser-induced leukoderma or vitiligo cases. Less frequent is laser-induced leukoderma or vitiligo caused by ablative and pigmented lesion procedures (13.3%) and in only one case (3.3%) a vascular laser procedure caused leukoderma or vitiligo.

The face and the legs were the two most affected body parts: 43.3% of the leukoderma or vitiligo lesions developed in the face and 26.7% on the legs. Moreover 10% of the leukoderma or vitiligo lesions developed on the arms and 6.7% on the axilla. In 56.7%, percent of the cases the leukoderma or vitiligo lesions developed 4–12 weeks after the treatment. Furthermore, in 26.7% of the cases, the lesions developed within 4 weeks and in 10% of the cases, it took more than 12 weeks before the leukoderma or vitiligo lesion occurred. For 6.7%, of the cases the information remained unknown.

**Fig. 2:**
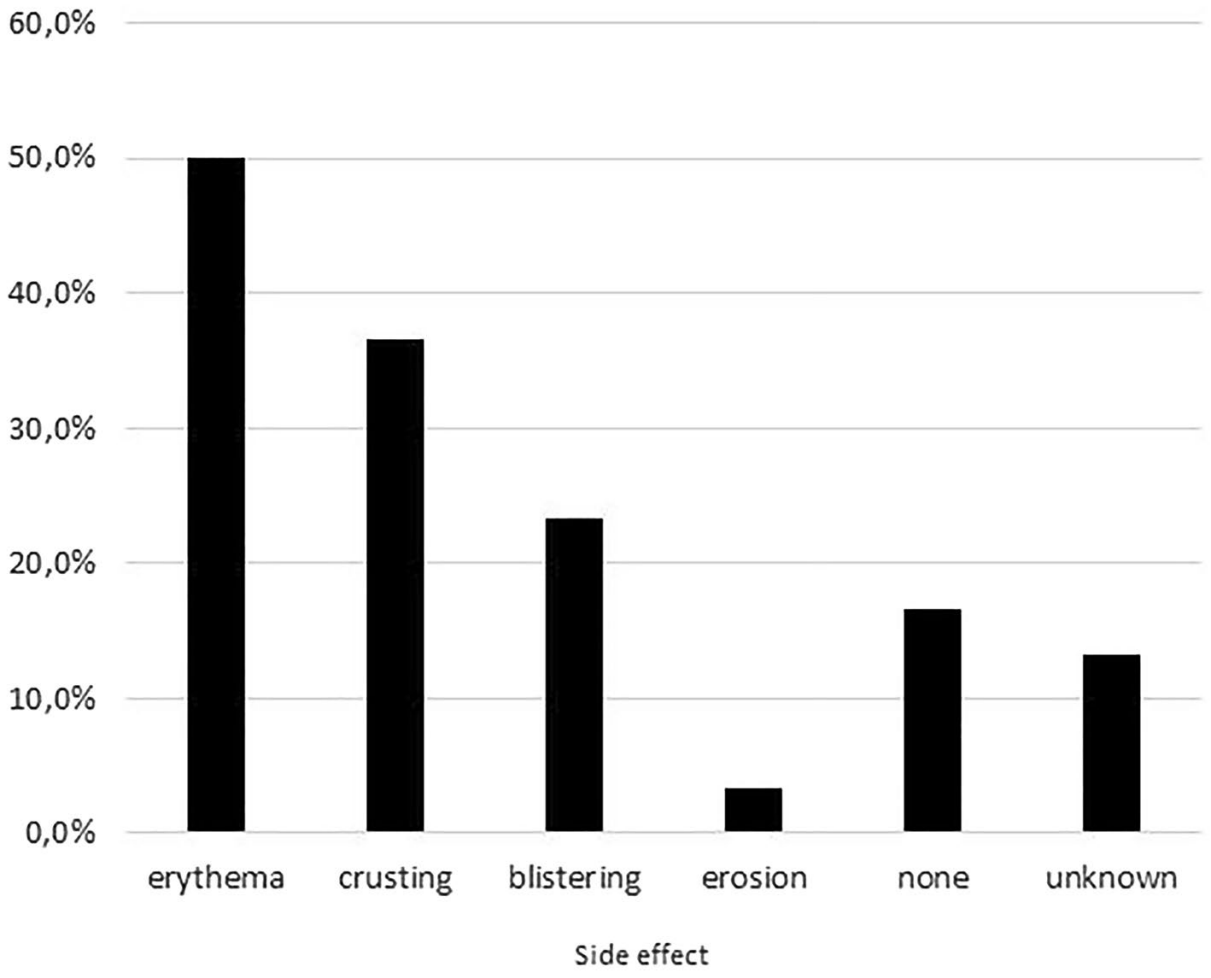
Side effects after the laser or IPL treatment

In Fig. 2, the reported side effects are presented. In 50% of the cases of laser-induced leukoderma or vitiligo erythema occurred, a normal skin reaction after laser or IPL treatment. Blistering, crusting, and erosion alone or combined with erythema occurred in 56.7% of the laser-induced leukoderma or vitiligo cases. In 23.3% of the cases blistering occurred. Furthermore, in 36.7% crusting and 3.3% erosion occurred after the laser or IPL treatment. Lastly, in 16.7% of the cases no skin reaction was visible and in 13.3%, this information remained unknown.

### Management approaches

**Fig. 3:**
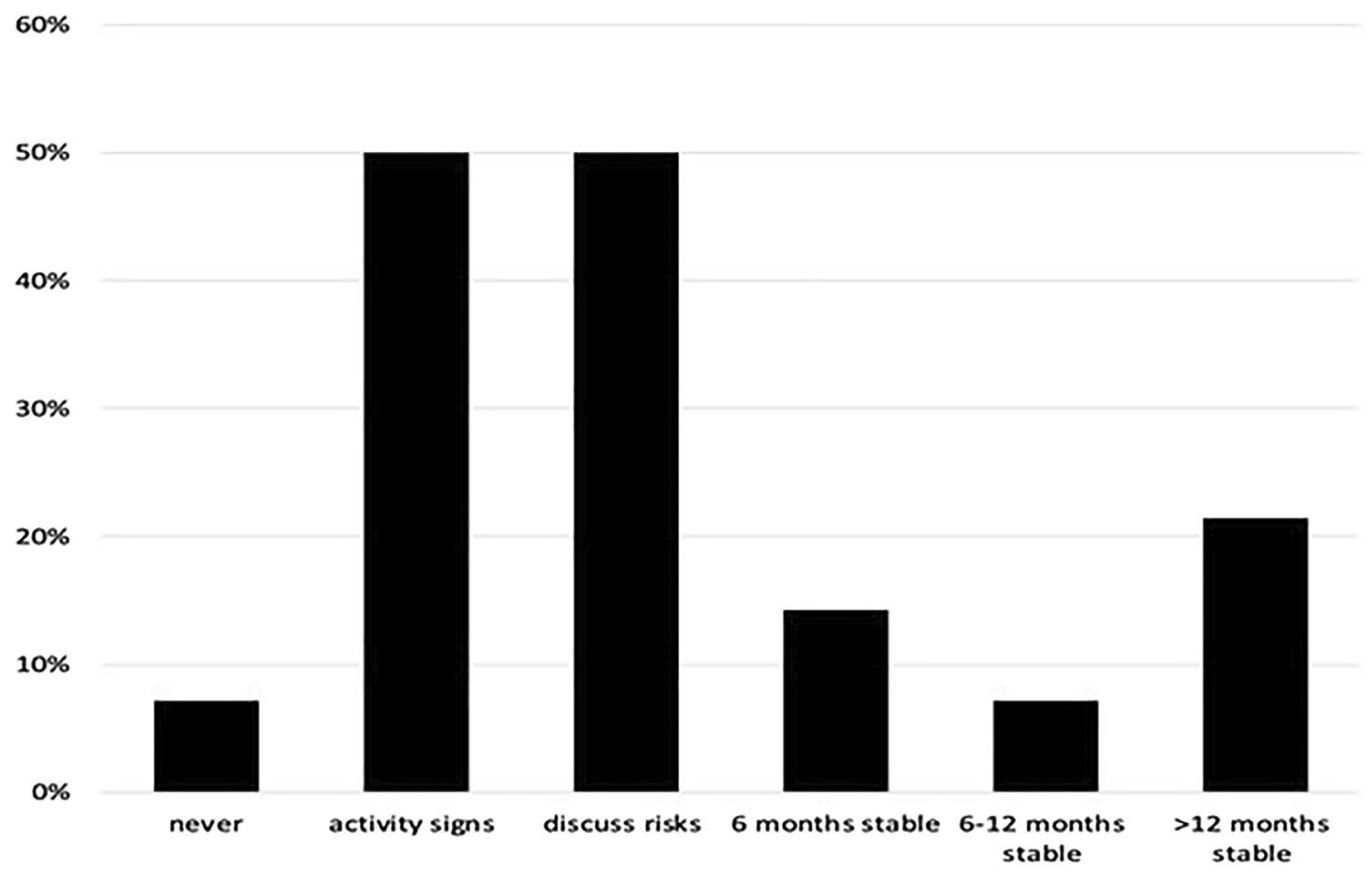
Experts’ apporach for vitiligo patients asking for a laser treatment

The participating dermatologists were asked for their approach regarding vitiligo patients who asked for a laser treatment. It was possible to give a combination of approaches. The results are shown in Fig. 3. One dermatologist did not perform any laser or IPL treatments in vitiligo patients regardless of activity signs, aggressiveness of laser settings or stability. 50% of the participating dermatologists based their advice on activity signs. Another 50% discuss the risks of the laser or IPL treatment with their vitiligo patients. Moreover, 43% of the dermatologists based their advice on the stability of the vitiligo. Specified, 14% advised laser or IPL therapy when the vitiligo is at least 6 months stable, 7% when vitiligo is stable for 6–12 months and 21% advised laser therapy when vitiligo was at least 12 months stable. Aggressiveness of the laser or IPL procedure (i.e. the risk for epidermal damage) was considered an important factor on which advice is based with 36% of the participating dermatologists stating this was a key consideration for them. Furthermore, one dermatologist added to the free text box to give tacrolimus after the treatment. All dermatologists believed that some restrictions and considerations were needed in those with vitiligo that desired laser or IPL treatments.

When considering activity signs as relevant for the risk of the laser treatment 57.1% of the dermatologists consider the Koebner phenomenon and confetti like lesions as relevant. Moreover, 50% of the dermatologists consider hypochromic borders relevant for the risk of the laser treatment.

## Discussion

The aim of this international survey study among vitiligo experts was to provide an estimation of the occurrence and related risk factors of laser/IPL-induced leukoderma or vitiligo.

This survey study demonstrates that laser/IPL induced leukoderma or vitiligo is relatively rare with only 30 cases out of a total of 11,300 vitiligo patients. This is in line with a recently published systematic review that identified only six cases, that laser/IPL-induced vitiligo is a rarely published phenomenon [6].

Unexpectedly, we observed that most of the cases involved patients without a medical history of vitiligo. Consequently, even when abstaining from treating patients with vitiligo, most cases could not have been prevented. Only 12 cases had a medical history of vitiligo. Moreover, in more than half of the patients with a medical history of vitiligo, the patients had a stable disease course of more than one year, which means that stability for 12 months does not guarantee a safe laser or IPL treatment. On the other hand, the finding of only seven stable vitiligo patients who developed new lesions after treatment from a total of 11,300 vitiligo patients might be regarded as an acceptable risk.

Epidermal damage such as blistering, crusting, and erosions was seen in 56.7% of the patients, suggesting that aggressive laser treatments increase the risk in the development of laser/IPL-induced leukoderma or vitiligo. This corresponds to previous research [5] and current dermatologic practice in which the Koebner phenomenon indicates both active vitiligo and a risk of developing vitiligo in a treated site. Caution is advised during laser or IPL treatments with aggressive settings. Furthermore test-spots prior to the regular treatment could indicate appropriate laser settings and decrease the risk for laser/IPL-induced leukoderma or vitiligo. Laser/IPL-induced leukoderma or vitiligo in patients that did not show epidermal damage could be explained by the occurrence of oxidative stress without epidermal damage.

Laser hair removal treatments were involved in 46.7% of the laser/IPL-induced leukoderma or vitiligo cases which reflects the large number of these procedures in laser practice. However, hypopigmentation is a well-known side effect of laser hair removal, especially in persons with higher skin types as melanin is targeted in laser hair removal [8] leading to thermally induced destruction of melanocytes [9] and potential induction of autoimmunity in genetically susceptible persons. The laser/IPL-induced leukoderma or vitiligo cases were mostly reported in patients with Fitzpatrick skin types 3 (46.7%) and 4 (43.3%), which corresponds with the distribution of the 11,300 vitiligo patients’ Fitzpatrick skin types 3 (46.6%) and 4 (34.7%). The majority of the lesions occurred on the face and the legs which again may reflect the large number of hair removal treatments of the face and legs.

Remarkably, when vitiligo patients ask for a laser treatment, vitiligo experts have different recommendations. One expert would never perform a (cosmetic) laser treatment in a vitiligo patient. Moreover, half of the participating dermatologists based their advice on activity signs. Interestingly, only 50–57.1% of the dermatologist consider hypochromic borders, the Koebner phenomenon, and confetti-like lesions as relevant activity signs for the risk of a laser treatment, although multiple studies showed that an active vitiligo and the Koebner phenomenon are related to higher depigmentation results with Q-switched (QS) nanosecond lasers [10, 11]. Moreover, all the three activity signs can be validated and reliably linked to disease activity by the Vitiligo Signs of Activity Score for physicians (VSAS) [12].

There are several limitations to this study. Firstly, the retrospective nature of this study means an inherent recall bias. Secondly, although the participating dermatologists of multiple countries on different continents provide a representation of the world-wide population, there is a selection bias, since seven participants are from European countries and all participants work in tertiary hospitals. Moreover, although all the participating dermatologists are experts in the field of vitiligo, it remains debatable whether the de novo cases are true vitiligo or laser/IPL-induced leukoderma. To minimize this bias we defined laser/IPL-induced vitiligo as a progressive depigmentation in a laser/IPL treated area (in patients with or without a history of vitiligo) and asked for the likelihood that the laser/IPL treatment caused the progressive depigmentation. However, we were not able to distinguish between laser/IPL-induced vitiligo and laser/IPL-induced leukoderma since there is no consensus about their definition yet. Furthermore, the lasers/IPL treatments were classified according to the purpose of the laser treatment, due to the fact that patients are most of the times unaware of the specific laser systems. Lastly, the incidence of laser/IPL-induced leukoderma or vitiligo in this study could be an overestimation, because the participating dermatologists are all specialized in vitiligo and lasers in their countries.

*In summary,* laser-induced leukoderma or vitiligo is an uncommon phenomenon. Remarkably, 60% of the reported cases did not have a medical history of vitiligo and more than half of the cases with a history of vitiligo had > 12 months of stable vitiligo, both suggesting it is difficult to prevent these cases. Moreover in 56.7% of the cases an unexpected complication occurred after the laser or IPL treatment. Advice for those with vitiligo who request a laser or IPL treatment should be based on activity signs, discussing the risks before treatment, and the stability of the vitiligo. Future research should be performed to provide a clear and concise guideline regarding laser or IPL-induced leukoderma and to distinguish between laser/IPL-induced leukoderma or vitiligo.

## Supplementary Information

Below is the link to the electronic supplementary material.

Supplementary file1 (DOCX 165 kb)

Supplementary file2 (DOCX 21 kb)

## Data Availability

The datasets generated during and/or analyzed during the current study are available from the corresponding author on reasonable request.

## References

[1] Ezzedine K, Eleftheriadou V, Whitton M, van Geel N (2015). Vitiligo Lancet.

[2] Thissen M, Westerhof W (1997). Laser treatment for further depigmentation in vitiligo. Int J Dermatol.

[3] Van Geel N, Speeckaert R, De Wolf J (2012). Clinical significance of Koebner phenomenon in vitiligo. Br J Dermatol.

[4] Van Geel N, Speeckaert R, Taieb A (2011). Koebner's phenomenon in vitiligo: European position paper. Pigment Cell Melanoma Res.

[5] van Geel N, Grine L, De Wispelaere P (2019). Clinical visible signs of disease activity in vitiligo: a systematic review and meta-analysis. J Eur Acad Dermatol Venereol.

[6] Post NF, Van Broekhoven NX, Bekkenk MW, Wolkerstorfer A (2022) Laser- and intense pulsed light (IPL)-induced vitiligo patches: a systematic review of the literature - short report. Lasers Med Sci. 10.1007/s10103-022-03582-4 35639193

[7] Sharma A, Minh Duc N (2021). A consensus-based checklist for reporting of survey studies (CROSS). J Gen Intern Med.

[8] Gan SD, Graber EM (2013). Laser hair removal: a review. Dermatol Surg.

[9] Lim SPR, Lanigan SW (2006). A review of the adverse effects of laser hair removal. Lasers Med Sci.

[10] Kim YJ, Chung BS, Choi KC (2001). Depigmentation therapy with Q-switched ruby laser after tanning in vitiligo universalis. Dermatol Surg.

[11] Van Geel N, Depaepe L, Speeckaert R (2015). Laser (755 nm) and cryotherapy as depigmentation treatments for vitiligo: a comparative study. J Eur Acad Dermatol Venereol.

[12] Van Geel N, Passeron T, Wolkerstorfer A (2020). Reliability and validity of the Vitiligo Signs of Activity Score (VSAS). Br J Dermatol.

