# Supplementary material for: Expert opinion about laser and intense pulsed light (IPL)-induced leukoderma or vitiligo: a cross-sectional survey study
Source: Arch Dermatol Res. 2023 Mar 25;315(8):2289–94. doi: 10.1007/s00403-023-02611-8 (PMC10462531; doi:10.1007/s00403-023-02611-8)
Supplement: Supplementary file 1 — Supplementary file1 (DOCX 165 kb) [file 403_2023_2611_MOESM1_ESM.docx]

# Supplementary file 1

Questionnaire - Laser induced vitiligo

This questionnaire is part of our research project into the possible causal relationship between laser or IPL treatment and the development of vitiligo (patches). Please note that we define laser induced vitiligo as progressive depigmentation in a laser treated area (in patients with or without a history of vitiligo). QSA laser treatment with the intention to induce depigmentation is excluded.

1. E-mailadres
2. We need your consent to process your answers to the questionnaire. Your data will only be used for research purposes and processed in accordance with the privacy statement. Do you give permission to process the answers and save these for 15 years?

Yes, I give permission

No, I do not consent

1. How many patients with vitiligo did you approximately encounter (face to face) over the past year?
2. How many of these patients were suspected for laser or IPL induced vitiligo?
   1. (scroll down to bottom of this page)

2

3

4

5

6

7

8

9

10

Patient 1*(this section can be filled in 10 times dependent on the amount of cases)*

Below you may provide information on the first patient that you suspect of laser or IPL induced vitiligo. The last question will give you the opportunity to provide information on more patients or to proceed to the end if this questionnaire.

1. How likely is it that the vitiligo lesion(s) in this patient were induced by laser or IPL therapy?

very likely likely

unlikely

very unlikely

1. Which type of procedure was involved?

Hair removal procedure Pigmented lesion procedure Vascular lesion procedure Ablative procedure

Fractional laser procedure

Skin rejuvenation

Anders:

1. Did the patient have a medical history of vitiligo prior to the treatment?

Yes No

1. If yes, was there a history of active/progressive vitiligo prior to the treatment?

Not applicable Unknown

Stable

Active in the last 6 months

Active in the last 6 to 12 months

Anders:

1. How long after the laser or IPL treatment did the vitiligo lesions develop?

0-4 weeks

4-12 weeks

More than 12 weeks

1. Where was the induced vitiligo lesion localized?
2. Were there any side effects to the skin visible after the laser or IPL treatment?

None

Erythema

Blistering

Crusting

Erosion

Ulceration

Unknown

Other:

1. Did you encounter more patients? (click yes if you previously indicated that more than 1 patient was suspected for laser or IPL induced vitiligo)

Yes

No

1. What is your approach for vitiligo patients asking for a laser treatment?

No restrictions

I discuss the risks of laser treatment to induce vitiligo

I advise not to do any laser treatments regardless of activity sign, aggressiveness of

laser settings or stability

Advise is based on how aggressive the laser treatment will be (i.e. how high the risk for

epidermal damage is)

I advise laser therapy when the vitiligo is at least 6 months stable

I advise laser therapy when the vitiligo is at least 6 to 12 months stable

I advise laser therapy when the vitiligo is more than 12 months stable

Advise is based on activity signs

Other:

1. Which activity signs do you consider relevant for your advice on the risks of laser treatment?

Pinpoint/ Confetti like depigmentation

Koebner phenomenon

Hypochromic borders

Not applicable

Other:

End

1. Thank you for your participation, if you have any additional comments please let us know.
