# Supplementary material for: Expert opinion about laser and intense pulsed light (IPL)-induced leukoderma or vitiligo: a cross-sectional survey study
Source: Arch Dermatol Res. 2023 Mar 25;315(8):2289–94. doi: 10.1007/s00403-023-02611-8 (PMC10462531; doi:10.1007/s00403-023-02611-8)
Supplement: Supplementary file 2 — Supplementary file2 (DOCX 21 kb) [file 403_2023_2611_MOESM2_ESM.docx]

**Checklist for Reporting Of Survey Studies (CROSS)**

| **Section/topic** | **Item** | **Item description** | **Reported on page #** |
| --- | --- | --- | --- |
| **Title and abstract** | | |  |
| Title and abstract | √ 1a | State the word “survey” along with a commonly used term in title or abstract to introduce the study’s design. **🡪 page 1, line 3** |  |
|  | √ 1b | Provide an informative summary in the abstract, covering background, objectives, methods, findings/results, interpretation/discussion, and conclusions. **🡪 page 2, line 30-42, page 3 line 1-16** |  |
| **Introduction** | | |  |
| Background | √ 2 | Provide a background about the rationale of study, what has been previously done, and why this survey is needed. **🡪 page 2, line 18-32** |  |
| Purpose/aim | √ 3 | Identify specific purposes, aims, goals, or objectives of the study. **🡪 page 2, line 31+32** |  |
| **Methods** | | |  |
| Study design | √ 4 | Specify the study design in the methods section with a commonly used term (e.g., cross-sectional or longitudinal). **🡪 page 3, line 35** |  |
|  | √ 5a | Describe the questionnaire (e.g., number of sections, number of questions, number and names of instruments used). **🡪 page 3, line 38-41** |  |
| Data collection methods | √ 5b | Describe all questionnaire instruments that were used in the survey to measure particular concepts. Report target population, reported validity and reliability information, scoring/classification procedure, and reference links (if any). **🡪 page 3, line 35-37** |  |
|  | √ 5c | Provide information on pretesting of the questionnaire, if performed (in the article or in an online supplement). Report the method of pretesting, number of times questionnaire was pre-tested, number and demographics of participants used for pretesting, and the level of similarity of demographics between pre-testing participants and sample population. **🡪 page 3, line 36-37** |  |
|  | √ 5d | Questionnaire if possible, should be fully provided (in the article, or as appendices or as an online supplement). **🡪 supplementary file 1** |  |
| Sample characteristics | √ 6a | Describe the study population (i.e., background, locations, eligibility criteria for participant inclusion in survey, exclusion criteria). **🡪 page 3, line 35-42, page 4, line 1-9** |  |
|  | √ 6b | Describe the sampling techniques used (e.g., single stage or multistage sampling, simple random sampling, stratified sampling, cluster sampling, convenience sampling). Specify the locations of sample participants whenever clustered sampling was applied. **🡪 page 3, line 12+13** |  |
|  | **N. A**. 6c | Provide information on sample size, along with details of sample size calculation. **🡪 page 4, line 13+14 (explains why n.a.)** |  |
|  | **N. A**. 6d | Describe how representative the sample is of the study population (or target population if possible), particularly for population-based surveys. |  |
| Survey  administration | √ 7a | Provide information on modes of questionnaire administration, including the type and number of contacts, the location where the survey was conducted (e.g., outpatient room or by use of online tools, such as SurveyMonkey). **🡪 page 3, line 38** |  |
|  | √ 7b | Provide information of survey’s time frame, such as periods of recruitment, exposure, and follow-up days. **🡪 page 4, line 9+10, line 13** |  |
|  | √ 7c | Provide information on the entry process:  **N. A.** –>For non-web-based surveys, provide approaches to minimize human error in data entry.  –>For web-based surveys, provide approaches to prevent “multiple participation” of participants. **🡪 page 4, line 22-24** |  |
| Study preparation | √ 8 | Describe any preparation process before conducting the survey (e.g., interviewers’ training process, advertising the survey). **🡪 page 4, line 21-22** |  |
| Ethical considerations | √ 9a | Provide information on ethical approval for the survey if obtained, including informed consent, institutional review board [IRB] approval, Helsinki declaration, and good clinical practice [GCP] declaration (as appropriate). **🡪 page 4, line 20-21** |  |
|  | √ 9b | Provide information about survey anonymity and confidentiality and describe what mechanisms were used to protect unauthorized access. **🡪 page 4, line 21-24** |  |
| Statistical  analysis | √ 10a | Describe statistical methods and analytical approach. Report the statistical software that was used for data analysis. **🡪 page 4, line 17-19** |  |
|  | **N. A.** 10b | Report any modification of variables used in the analysis, along with reference (if available). |  |
|  | √ 10c | Report details about how missing data was handled. Include rate of missing items, missing data mechanism (i.e., missing completely at random [MCAR], missing at random [MAR] or missing not at random [MNAR]) and methods used to deal with missing data (e.g., multiple imputation). **🡪 page 4, line 12** |  |
|  | √ 10d | State how non-response error was addressed. **🡪 page 4, line 9+10** |  |
|  | **N. A.** 10e | For longitudinal surveys, state how loss to follow-up was addressed. |  |
|  | N. A. 10f | Indicate whether any methods such as weighting of items or propensity scores have been used to adjust for non-representativeness of the sample. |  |
|  | **N. A**. 10g | Describe any sensitivity analysis conducted. |  |
| **Results** | | |  |
| Respondent characteristics | √ 11a | Report numbers of individuals at each stage of the study. Consider using a flow diagram, if possible. **🡪 page 4, line 26** |  |
|  | √ 11b | Provide reasons for non-participation at each stage, if possible. **🡪 page 4, line 26+27** |  |
|  | √ 11c | Report response rate, present the definition of response rate or the formula used to calculate response rate. **🡪 page 4, line 26** |  |
|  | √ 11d | Provide information to define how unique visitors are determined. Report number of unique visitors along with relevant proportions (e.g., view proportion, participation proportion, completion proportion). **🡪 page 4, line 22-24** |  |
| Descriptive  results | √ 12 | Provide characteristics of study participants, as well as information on potential confounders and assessed outcomes. **🡪 page 3, line 35-37 + page 4, line 26-29** |  |
| Main findings | **N. A.** 13a | Give unadjusted estimates and, if applicable, confounder-adjusted estimates along with 95% confidence intervals and p-values. |  |
|  | **N. A.** 13b | For multivariable analysis, provide information on the model building process, model fit statistics, and model assumptions (as appropriate). |  |
|  | **N. A.** 13c | Provide details about any sensitivity analysis performed. If there are considerable amount of missing data, report sensitivity analyses comparing the results of complete cases with that of the imputed dataset (if possible). |  |
| **Discussion** | | |  |
| Limitations | √ 14 | Discuss the limitations of the study, considering sources of potential biases and imprecisions, such as non-representativeness of sample, study design, important uncontrolled confounders. **🡪 page 6, line 37-45, page 7, line 1+2** |  |
| Interpretations | √ 15 | Give a cautious overall interpretation of results, based on potential biases and imprecisions and suggest areas for future research. **🡪 page 6, line 1-36, page 7, line 4-10** |  |
| Generalizability | √ 16 | Discuss the external validity of the results. **🡪 page 6, line 1-36** |  |
| **Other sections** | | |  |
| Role of funding source | √ 17 | State whether any funding organization has had any roles in the survey’s design, implementation, and analysis. **🡪 page 1, line 37** |  |
| Conflict of interest | √ 18 | Declare any potential conflict of interest**. 🡪 page 2, line 1** |  |
| Acknowledgements | √ 19 | Provide names of organizations/persons that are acknowledged along with their contribution to the research. **🡪 page 1, line 5-23, page 6, line 12+13** |  |
